# Application of Two-Part Statistics for Comparison of Sequence Variant Counts

**DOI:** 10.1371/journal.pone.0020296

**Published:** 2011-05-23

**Authors:** Brandie D. Wagner, Charles E. Robertson, J. Kirk Harris

**Affiliations:** 1 Department of Biostatistics and Informatics, Colorado School of Public Health, University of Colorado Denver, Aurora, Colorado, United States of America; 2 Department of Molecular, Cellular and Developmental Biology, University of Colorado Boulder, Colorado, United States of America; 3 Department of Pediatrics, School of Medicine, University of Colorado Denver, Aurora, Colorado, United States of America; University of New Orleans, United States of America

## Abstract

Investigation of microbial communities, particularly human associated communities, is significantly enhanced by the vast amounts of sequence data produced by high throughput sequencing technologies. However, these data create high-dimensional complex data sets that consist of a large proportion of zeros, non-negative skewed counts, and frequently, limited number of samples. These features distinguish sequence data from other forms of high-dimensional data, and are not adequately addressed by statistical approaches in common use. Ultimately, medical studies may identify targeted interventions or treatments, but lack of analytic tools for feature selection and identification of taxa responsible for differences between groups, is hindering advancement. The objective of this paper is to examine the application of a two-part statistic to identify taxa that differ between two groups. The advantages of the two-part statistic over common statistical tests applied to sequence count datasets are discussed. Results from the t-test, the Wilcoxon test, and the two-part test are compared using sequence counts from microbial ecology studies in cystic fibrosis and from cenote samples. We show superior performance of the two-part statistic for analysis of sequence data. The improved performance in microbial ecology studies was independent of study type and sequence technology used.

## Introduction

Analysis of sequence variants, particularly the small subunit ribosomal RNA gene (SSU-rRNA), is widely used to examine microbial ecology. The concept of the microbiome, the genetic content of all microbes present in a community, was articulated to promote the study of microbial ecology of the human body [1]. Sequencing methods are used to generate data in several areas of human health and across diverse ecological studies. This DNA based method for bacterial identification has many advantages over culture-based methods and provides the ability to identify organisms without a priori knowledge of the community present [2], [3]. Typical data generated from a microbial ecology study consist of SSU-rRNA gene sequence variant counts. These variants serve as a proxy for the diversity and relative abundance of the microbial populations in the community. Sequences are classified based on relationship to exemplar sequences, which provides taxonomic information about the organism that contributed the template DNA [4].

Microbiome studies have been designed to compare bacterial communities across groups, but the majority of studies have not focused on methods that formally identify statistically significant differences between groups. The ultimate goal of microbial ecology studies is to understand the community constituents that perform particular functions. The human microbiome project endeavors to apply this to human associated communities in order to identify taxa that either adversely affect, or promote, health. A first step towards this goal will require researchers to extend beyond the description of which taxa are present and perform analyses capable of identifying important taxa. This move toward selection of informative taxa, which vary across disease groups, for refined study will identify targets for intervention or taxa which are helpful for prognostic or diagnostic purposes. This same concept of feature selection is employed in microarray studies and we propose the application of a similar approach to microbiome data.

As with microarray data, sequence data are high-dimensional but with added complexity. Instead of the lognormal continuous values obtained in microarray data, sequence data consist of non-negative, highly skewed sequence counts with a large number of zeros. The number of zeros in the dataset is a direct result of the combination of sequence counts from different communities. The samples within a group will provide unique taxa, which require insertion of zeros in all other samples within the other group. There is a need for methods to compare the sequence counts between different disease groups which can handle the specific features of the data. A further constraint is sample size, which is relatively small in many studies, where the asymptotic assumptions are not reasonable. Limited samples are often the result of difficulty obtaining human samples, which constrains the size of studies due to the expense of patient recruitment and sample collection. Environmental studies are still largely scaled to clone-based sequencing limitations. The availability of indexed (barcoded) libraries, sequenced by high throughput technologies, fundamentally changes the design constraints of sequence-based microbial ecology studies. Short sequences are a concern in environmental studies, but access to long read platforms is increasingly limited due to cost per base.

The unique characteristics of sequence count data are not adequately addressed by standard statistical approaches used to compare variables across groups such as t-tests and the nonparametric approaches that compare ranks. To identify taxa that differ between two groups, we propose the use of a two-part statistic, as it is capable of handling the complexities in the distribution of sequence count data. Application of this approach to other data types, particularly microarray data analysis [5], [6], has previously been described in the literature.

## Methods

### Motivating examples

Two datasets were used to demonstrate the utility of the two-part approach. One study involves clinical samples and Sanger sequence data, and the other, environmental samples and 454 pyrosequence data. These examples were selected to cover the two primary modes of sequence data acquisition for both clinically relevant microbiome and environmental ecology examples. The clinical dataset is from a cystic fibrosis (CF) research study performed at The Children's Hospital of Denver. The contrast in this example is between CF sputum samples obtained during active disease (acute pulmonary exacerbation, n = 16) and sputum obtained from healthy controls (n = 10) by induction [7]. Bacteria were identified by culture-independent methods based on sequence of the SSU-rRNA. The environmental dataset was obtained from two cenote sites in Mexico [8], [9]. The sites were sampled extensively, and 49 and 60 amplicon libraries were contrasted between the two sites.

### Ethics Statement

All human specimens were collected under approved protocols by the Colorado Multiple Institutional Review Board (COMIRB). Written informed consent and HIPPA Authorization were obtained from all participants over the age of 17 years or from parents or legal guardians of participants younger than 18 years. Assent was obtained from all participants under 18 years.

### Sequence Analysis

#### Sanger Data

Contiguous small subunit ribosomal RNA sequences from multiple sequencing reactions were assembled using the program Xplorseq 2.0 [10] and compared to a database of well-curated sequences of isolates derived from Silva 93 [11] using BLAST [12] to determine their approximate phylogenetic relationships. Sequences were aligned using NAST [13] and parsimony inserted into a database provided by Greengenes [14] compatible with the ARB software package [15]. Each sequence was then assigned a taxa name based on the phylogenetic placement in the Greengenes guide tree. Chimeras were detected from long-branch lengths within the phylogenetic tree and confirmed by comparing the best match by BLAST for each end of the sequence. Sequences that were considered chimeras were excluded.

#### 454 Data

Sequence data was assigned to the appropriate samples based on the barcode included prior to sequencing with the software package Bartab [10]. Sequence quality was checked using ChimeraSlayer [16], correct bacterial rRNA secondary structure with Infernal [17], and identification as bacterial using the RDP Classifier [18]. The taxonomy lines generated by the RDP Classifier were used to construct the sequence count data examined.

### Description of data

Within the CF dataset, 175 different species level taxa were identified. *Veillonella dispar*, *Granulicatella adiacens* and *Streptococcus sanguis* are used throughout to represent the range of zero count proportions that characterize the full dataset. The environmental cenote dataset resulted in sequence counts for 827 genus level taxa. *Desulfobacca, Chlorobium and Dehalogenimonas* had similar proportions of zeros, and were evaluated in depth to highlight the differences between the three methods. The total number of sequences varies across samples, and requires normalization. Thus, relative abundance, the percent of the total number of sequences obtained for each taxa within a sample, was used in lieu of the raw sequence counts.

### Statistical Analyses

With a large enough sample size, the application of a t-test to skewed counts where data do not represent a continuum of values is appropriate. In the case where a sample size is not sufficiently large for the means to approximate a normal distribution, the Wilcoxon rank-based approaches are usually recommended. However, neither of these approaches is suitable where there is a large proportion of zeros, as it would result in either a deflated mean, or in the case of a rank based approach, a large number of ties, which reduces power [19], [20]. Neither approach capitalizes on the presence/absence information contained in the proportion of zero counts. An alternative is the two-part statistic, successfully used in similar applications [5], [19], [21], that is proposed here for analysis of sequence count data.

### Two-part statistics

In this approach, the test statistic is the sum of two squared statistics, one comparing the proportion of zeros and one comparing the mean or median of the non-zero values. For application to sequence count data with two independent groups, we propose the use of a two-proportion Z- test and the Wilcoxon rank sum test applied to the non-zero counts [19], [22]. More specifically, the two-proportion Z-test is used to compare the proportion of the non-zero counts and is calculated by the following equation:
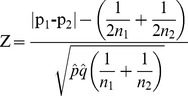
(1)where n_1_ and n_2_ are the number of total observations in group 1 and 2, respectively, the number and proportion of non-zero counts in each group are denoted by m_1_, p_1_ and m_2_, p_2_, and 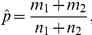 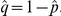


For the second part of our statistic, we use the Wilcoxon rank sum test to compare the medians of the non-zero counts. We use the Wilcoxon test, rather than a t-test or the Kolmogorov-Smirnov, because nonparametric tests based on ranks are more appropriate for skewed data such as sequence counts within small sample sizes. To calculate the ranks, first the data from the two groups are combined and the values across both groups are ranked, the average rank is assigned when there are tied values. The following test statistic is used to compare the non-zero relative abundance values across two independent groups [23]:
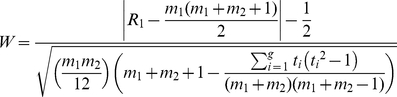
(1)where R_1_ is the sum of the ranks in group 1, t_i_ is the number of observations with the same value in the i-th tied group, and g is the number of tied groups. The variables m_1_ and m_2_ are the non-zero counts in each group as in the Z-test described above.

The normal approximation of this test statistic is appropriate when both m_1_ and m_2_ are greater than 10 and the underlying distribution is continuous. When there are no ties within the non-zero counts, the last term in the denominator reduces to zero.

For the extreme cases where there are no zero counts we set Z = 0 or when there are only zero counts in one group we set W = 0. The resulting two-sample test statistic is X2 = Z^2^+W^2^ which is asymptotically distributed χ^2^ with 2 degrees of freedom (df) [22]. This statistic tests the null hypothesis that the proportion of zero values and the location parameter describing the distribution of the non-zero values is equal across the groups. This statistic reaches statistical significance whenever either the proportion of zeros or the median of non-zero values differs substantially between groups. If the study design consists of paired observations, rather than independent groups, a similar approach using McNemar's test for paired proportions can be combined with the Wilcoxon Signed Rank Sum test as previously described [24].

## Results

**Figure 1:**
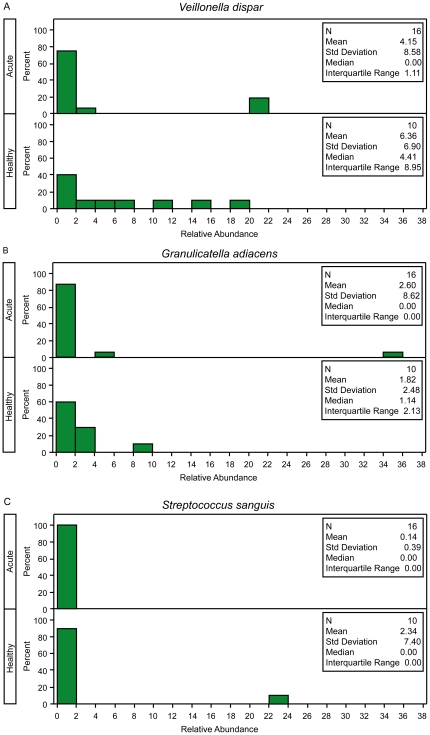
Distribution of relative abundance measures for each taxa across both disease groups. This figure displays histograms for the following three taxa chosen from the CF study to represent the range of proportion of zeros present in the dataset **A** *Veillonella dispar* **B** *Granulicatella adiacens* **C** *Streptococcus sanguis.*

**Figure 2:**
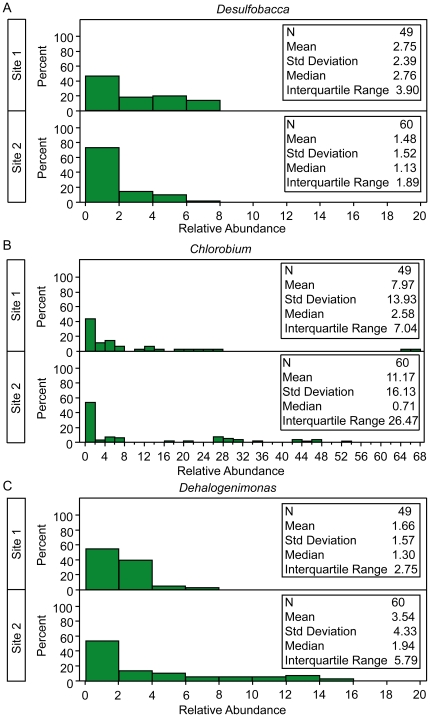
Distribution of relative abundance measures for each taxa between cenote sites. Histograms representing the relative abundance of the three taxa selected to represent the cenote sites **A** *Desulfobacca* **B** *Chlorobium* **C** *Dehalogenimonas.* These taxa were chosen from the cenote study to represent the differences in the distributions when the performance of the statistical approaches differed.

To compare the two-part statistic with the t-test and the Wilcoxon rank sum test, three taxa from each motivating dataset were selected. The selection was performed to demonstrate the performance of the three methods under different scenarios. In the CF study, the taxa were selected to represent the range of zero proportions in the distributions (Figure 1), and the environmental taxa were selected to investigate the distributional properties of the data when the results obtained across the three methods differed (Figure 2).

**Figure 3:**
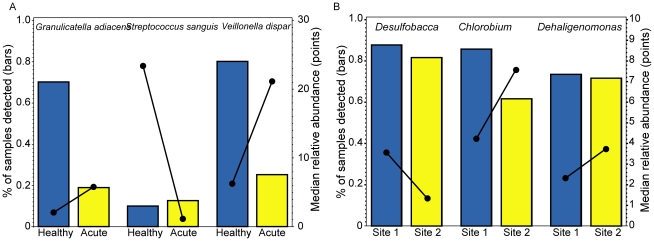
Displays the two components of the distribution for each taxa. The proportion of zero counts (bars) and the median of the non-zero counts (points) are displayed for the three taxa across both groups for **A** the CF dataset and **B** the cenote dataset.

For the CF study, *Veillonella dispar* had a small percentage of zero counts (54%) across the groups, *Granulicatella adiacens* (62%) was intermediate and *Streptococcus sanguis* had a high percentage at 89%. *Veillonella dispar* and *Granulicatella adiacens* are more often present in healthy samples compared to the acute group but when detected, the relative abundance in the healthy group is lower than the acute group (Figure 3a). *Streptococcus sanguis* is detected in similar proportions across the two groups, however, when it is detected, the relative abundance in the healthy group is much larger than the acute group. The taxa selected from the cenote study, *Desulfobacca, Chlorobium* and *Dehalogenimonas*, had similar percentages of zero counts overall (16% – 28%) and these percentages were comparable across both cenote sites for *Dehalogenimonas* and *Desulfobacca* (Figure 3b). However, *Chlorobium* had differing proportions of zero counts between sites (38% vs 14%). All three taxa from the cenote study had some difference in the median of the non-zero counts.

For the six taxa, a t-test, non-parametric Wilcoxon test and the two-part test, as described earlier, were used to compare the acute and healthy groups from the CF study and two sites from the cenote study (Table 1). For the majority of the examples, there is a large difference between the results obtained with a t-test and the two-part or Wilcoxon tests. For the comparisons of *Granulicatella adiacens* and *Veillonella dispar*, the t-test results are non-significant due to the increase in both the proportion of zeros and the median of the non-zero counts for the acute group compared to the healthy group. In this case, these two parameters are inversely related, thereby canceling each other out when a mean is calculated. This relationship is easily accommodated by the two-part test. For *Streptococcus sanguis*, only one sample in the healthy group was non-zero. Therefore, the non-significant two-part statistic is testing the slight difference in the proportions, whereas the t-test has a smaller p-value because the single non-zero outlier inflates the mean in the healthy group. For the cenote data, the *Desulfobacca* proportion of non-zeros and the non-zero counts were increased in the first site compared to the second, resulting in relative agreement across the three methods, although the two-part test was more conservative. Lastly, *Dehalogenimonas* was considered to have a highly significant difference when using the t-test (due to the non-normal distribution), non-significance using the Wilcoxon (likely due the high number of tied ranks) and a marginal significance using the two-part test.

**Table 1:** Comparison of results from two group comparison.

	T-testp-value	Wilcoxonp-value	Two-partp-value
***CF study***			
*Veillonella dispar*	0.50*	0.07**	0.02***
*Granulicatella adiacens*	0.74*	0.04***	0.05***
*Streptococcus sanguis*	0.37*	0.96**	0.75***
***Cenote study***			
*Desulfobacca*	<0.01***	0.01***	0.03**
*Chlorobium*	0.27**	0.44**	<0.01***
*Dehalogenimonas*	<0.01**	0.22**	0.08***

*Distribution for taxa includes an outlier which results in incorrect inferences.

**Assumptions of the test are not optimal given the distribution of the taxa (i.e., skewness, large proportion of zeros or power).

***Most optimal approach, given the distribution of the taxa.

**Figure 4:**
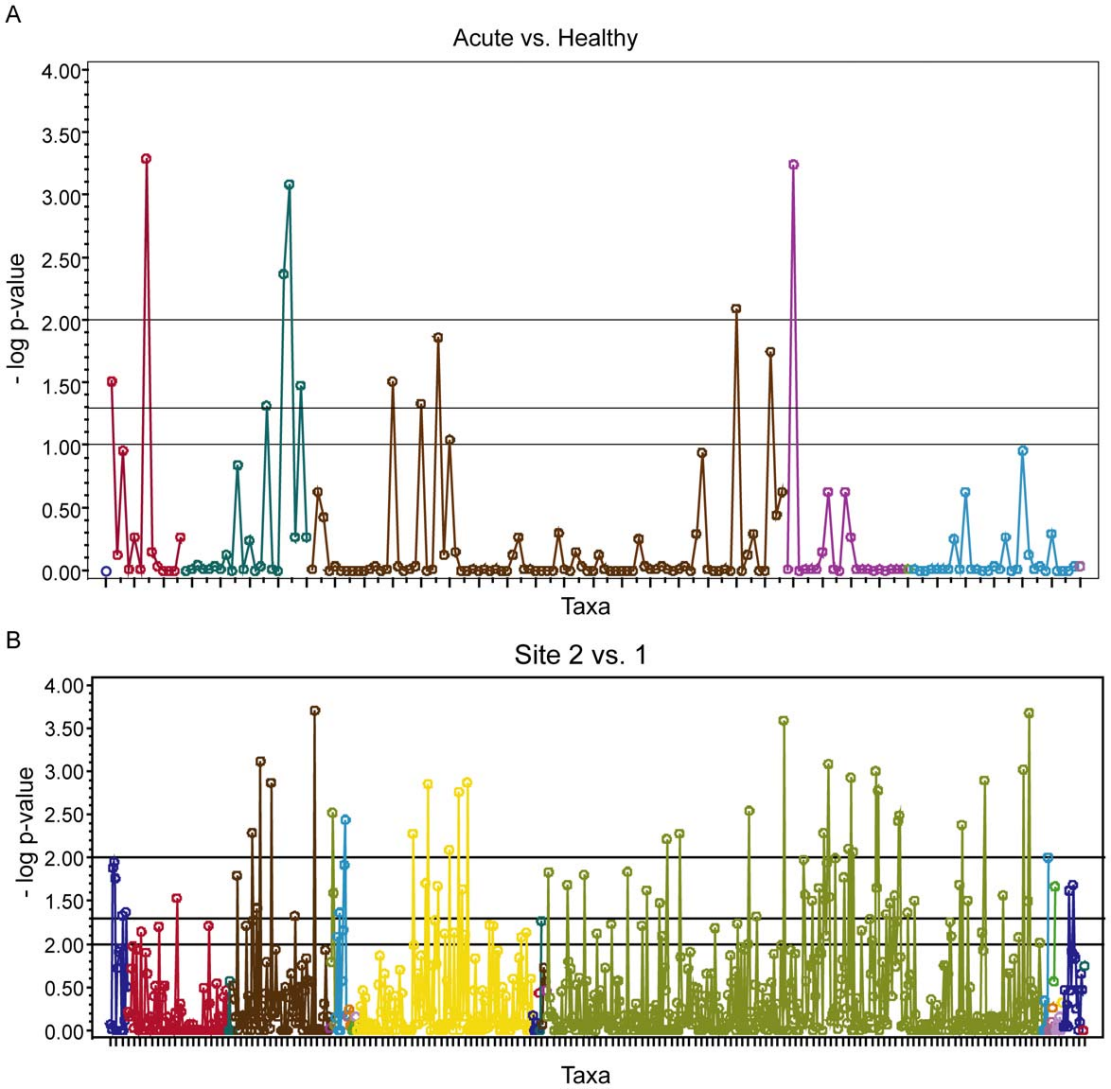
Manhattan plots displaying the results from all two-part tests across all taxa. The y-axis displays the negative log of the p-value, hence higher values indicate increased statistical significance. Reference lines are included to designate the usual critical values. The Manhattan plot is ordered by taxonomy line and the colors correspond to different phyla. **A** There were 12 species level taxa with p-values <0.05 identified in the CF study and **B** 79 genus level taxa were identified in the cenote study.

There were a total of 175 species level and 827 genus level taxa detected in the CF and cenote examples, respectively. To demonstrate the performance of feature selection, the two-part statistics were calculated separately on each taxa to compare the relative abundance between the two groups. A Manhattan plot, commonly used in genetic studies, was used to display the magnitude of the p-values for each comparison (Figure 4) with the taxa ordered by taxonomy line, and color-coded by phylum. This plot indicates that 12 of the 175 species level taxa, in the CF study, and 79 of the 827 genus level taxa, from the cenote samples, had statistically significant differences in relative abundance between the two groups (p<0.05). This plot can aid in feature selection and provides information on the number of potentially informative taxa within each phylum.

## Discussion

Here, we describe the distributions of the microbial sequence counts observed in two studies of the bacterial differences between two groups of samples. The distributions of the relative abundance variables are highly skewed, non-negative and have a large proportion of zeros, for which commonly used statistical approaches may not be appropriate. Three specific taxa from each study were presented in detail to demonstrate the performance of each approach. Based on this analysis, we show that the application of two-part tests provide more information about sequence count data compared to t-tests and Wilcoxon tests. The Wilcoxon and two-part tests produce similar results when there are smaller proportions of zeros in both groups, but as these proportions increase, the Wilcoxon test is less powerful due to the higher number of tied ranks.

In ecological research, count data with a large proportion of zeros is routinely encountered [25], [26], [27]. In fact, in this case, the large proportion of zeros is intrinsic to the creation of the dataset rather than the data generating process itself. The dataset contains sequence counts for taxa that were observed in at least one sample, if a particular taxon was not observed in a sample it is given a zero value. Therefore, when comparing sequence counts across two diverse groups with differences in the presence/absence of taxa, a large numbers of zero counts are expected. For this reason, it is likely that similar distributions are also encountered in other related sequencing applications such as allele frequency. Moreover, application of the two-part test proposed here is not restricted to sequence count data from microbial ecology studies.

The two-part statistic provides an analytic option for sequence count data due to the unique features observed, mainly, data which is non-normally distributed, high dimensional and contains a large proportion of zeros. Further, it performs an explicit test of both the proportion of samples that contain particular taxa and, simultaneously, the relative abundance between two groups. This approach overcomes limitations in other methods like the t- test, which is affected by outliers, and the Wilcoxon rank sum test that accommodates non-normality but loses power as the number of tied ranks, caused by the large number of zero counts, increases.

It has previously been shown that the two-part tests perform better than the other commonly used tests when the group with the larger proportion of zeros also has the larger mean, as demonstrated by the *Granulicatella adiacens*, *Veillonella dispar* and *Chlorobium* examples. If the opposite holds true then the two-part tests have somewhat reduced power with respect to the commonly used methods [19]. However, the application of the two-part test remains advantageous given the interest in simultaneously comparing the presence/absence and the mean quantities of taxa. Lachenbruch [19] provides an empirical simulation study which investigated and compared the power and type I error rates of the two-part test with the single degree of freedom tests considered here.

For large samples, the two parts of the two-part statistic (Z and W) are independent under the assumptions of independent errors of both parts of the test [28]. However, for smaller studies, where the distributional approximations of the two-part test are not reasonably assumed, extension of this method to a permutation based test [5] is warranted to more accurately estimate a corresponding p-value. In any case, the tests can be ranked based on the chi-squared statistic to similarly perform feature selection in the event a satisfactory approach to calculating a p-value cannot be obtained. The issue of multiple comparisons from application of the two-part test to each individual taxa was not addressed here. The expansion of the two-part test to a more general permutation based test will accommodate the permutation-based multiple comparisons adjustments previously applied to microarray studies. These approaches have the advantage to account for both the correlation between taxa and the association induced by the relative abundance calculation [29], [30], [31]. Additionally, for more complex study designs, the authors are developing zero-inflated models which others have advocated for this type of data [32] and are useful for generalizations which include ANOVAs, addition of covariates and repeated measures but which require adaptation and guidelines for high-dimensional applications.

To date, there is a fundamental lack of development and investigation of statistical methods appropriate for integrated sequence and metadata that resulted in an analysis bottleneck and backlog of potentially informative studies [33], [34], [35]. There are several published contentions that human microbiome research “lacks the range of computational tools necessary to analyze these sequences in sufficient detail” [36]. It is also recognized that interpretation of the available sequence data will require integration with relevant environmental, epidemiological and clinical data [33], [36]. The commonly used statistical methods applied in this area are intended for the calculation of global ecological parameters and the description of bacterial communities. These methods are not meant to address more focused questions related to specific taxa. The departure from more general inquiries about the overall community differences to analyses that focus on specific taxa is likely where the greatest advancement in knowledge of the human microbiome will come from. This transition is apparent in recent publications [37], [38]. To further proceed in this direction, we have proposed an initial strategy for comparisons between two groups and have shown it is appropriate for the specific attributes of microbiome data, irrespective of sample type, phylogenetic level and sequencing technology.
